# Daratumumab for relapsed refractory immune thrombotic thrombocytopenic purpura: initial response and long-term follow-up

**DOI:** 10.1016/j.rpth.2026.103405

**Published:** 2026-03-04

**Authors:** Roman Schimmer, Jana van den Berg, Marissa Schraner, Alice Trinchero, Andreas Holbro, Johanna A. Kremer Hovinga, Jan-Dirk Studt

**Affiliations:** 1 Department of Medical Oncology and Hematology, University of Zürich and University Hospital Zürich, Zürich, Switzerland; 2 Division of Hematology, University Hospital Basel, Basel, Switzerland; 3 Department of Hematology and Central Hematological Laboratory, Bern University Hospital, University of Bern, Bern, Switzerland; 4 Department for BioMedical Research, University of Bern, Bern, Switzerland

**Keywords:** daratumumab, recurrence, thrombotic microangiopathy, thrombotic thrombocytopenic purpura

## Abstract

**Background:**

Management of patients with refractory or frequently relapsing (r/r) immune thrombotic thrombocytopenic purpura (iTTP) remains challenging, with no established consensus on optimal therapeutic strategies. In recent years, plasma-cell depletion with daratumumab, a monoclonal antibody against CD38, has been used successfully for treatment-resistant patients with various autoimmune diseases, including iTTP, although long-term data remain limited.

**Objectives:**

To assess the efficacy, durability, and safety of daratumumab in patients with r/r iTTP.

**Methods:**

We retrospectively analyzed 8 daratumumab treatment episodes in 5 patients with r/r iTTP, treated at 3 Swiss tertiary centers, with a median follow-up after daratumumab of 44 months (range, 34-65 months). Serial a disintegrin and metalloproteinase with thrombospondin type 1 motif 13 (ADAMTS-13) activity and inhibitor levels, clinical outcomes, and adverse events were assessed.

**Results:**

Daratumumab induced rapid and robust ADAMTS-13 recovery in 7 of 8 episodes. All responding patients achieved complete ADAMTS-13 remission within 1 and 3 weeks. Mean ADAMTS-13 relapse-free survival following daratumumab was 32 months, with 7 of 8 episodes remained in complete ADAMTS-13 remission at 12 months. Nonetheless, ADAMTS-13 relapse occurred in 4 of 5 patients during long-term follow-up. Two patients experienced mild to moderate infusion-related adverse reactions.

**Conclusion:**

Our findings support daratumumab as an effective and well-tolerated therapeutic option for patients with r/r iTTP, particularly those unresponsive to rituximab and other immunosuppressants.

## Essentials

• Relapsed/refractory immune thrombotic thrombocytopenic purpura lacks clear treatments.
• We studied daratumumab use in 5 Swiss patients with relapsed or refractory immune thrombotic thrombocytopenic purpura.
• Daratumumab rapidly restored a disintegrin and metalloproteinase with thrombospondin type 1 motif 13 (ADAMTS-13) activity in most treatment episodes.
• Responses were durable and treatment was generally well tolerated.

## 1 Introduction

Immune thrombotic thrombocytopenic purpura (iTTP) is a life-threatening thrombotic microangiopathy caused by severe deficiency of the von Willebrand factor (VWF)–cleaving protease a disintegrin and metalloproteinase with thrombospondin type 1 motif 13 (ADAMTS-13), due to autoantibodies inhibiting ADAMTS-13 activity or accelerating its clearance. ADAMTS-13 deficiency impairs the cleavage of ultralarge VWF, promoting excessive VWF–platelet interaction and microvascular thrombus formation. This results in thrombocytopenia, microangiopathic hemolytic anemia, and tissue ischemia [1]. Prior to the advent of effective therapies, mortality of iTTP exceeded 90%; even today, it remains as high as 6% in experienced centers [2,3].

The current standard of care for acute iTTP rests on 3 pillars: daily therapeutic plasma exchange; caplacizumab, an anti-VWF nanobody; and immunosuppression with glucocorticoids and the anti-CD20 antibody rituximab [[4], [5], [6]]. While the addition of rituximab has reduced the risk of relapse, it does not provide an immediate therapeutic effect and fails to normalize ADAMTS-13 activity in ∼15% of cases [7]. Relapse after a first episode still occurs in at least 30% of patients [6,8].

Some patients show a frequently relapsing course or are resistant to treatment (persistent ADAMTS-13 deficiency and inhibitor), termed relapsing or refractory or frequently relapsing (r/r) iTTP. Management remains challenging due to an incomplete understanding of the underlying pathophysiology and the lack of clearly defined immunosuppressive strategies. Reported therapies include mycophenolate [9], azathioprine [10], cyclophosphamide [11], cyclosporine [12], bortezomib [13], obinutuzumab [14], and splenectomy [15]. However, several of these are associated with significant side effects, and clinical data remain scarce.

A proposed mechanism underlying r/r iTTP is the persistence or reemergence of autoantibodies to ADAMTS-13, driven by pathogenic ADAMTS-13–specific B cells, most likely plasmablasts and long-lived plasma cells [16]. These cell populations have also been implicated in the pathogenesis of other autoimmune diseases [17]. Unlike naïve and memory B cells, plasmablasts and plasma cells do not express CD20 and are therefore unaffected by anti-CD20 therapy. They, however, express CD38, which is targeted by daratumumab, a monoclonal antibody approved for multiple myeloma. Daratumumab has demonstrated efficacy in several treatment-resistant or refractory antibody-mediated autoimmune disorders and is generally well tolerated [18].

Emerging evidence suggests that daratumumab may be effective for treating r/r iTTP [[19], [20], [21]]. After describing the first successful application of daratumumab in 2 patients with r/r iTTP [19], we present real-world data from a Swiss multicenter cohort, focusing on long-term follow-up, treatment durability, and retreatment outcomes.

## 2 Methods

### 2.1 Patients and treatment

This was a retrospective multicenter cohort study. Adult patients diagnosed with iTTP and treated with daratumumab in Switzerland were eligible for this study. Cases were identified through a survey of members of the Swiss Society of Hematology Working Party Hemostasis, the ADAMTS-13 reference laboratory database at Bern University Hospital, and hospital International Classification of Diseases–coded discharge records.

We identified 8 daratumumab treatment episodes in 5 adult patients with r/r iTTP across 3 Swiss tertiary centers (university hospitals of Zürich, Basel, and Bern), all of whom participated in this study. Data collected from the patient charts included clinical course, treatment, adverse reactions, and serial measurements of ADAMTS-13 activity and inhibitor.

Initial management of acute iTTP followed current international guidelines [4]. All patients had received at least 1 prior course of rituximab at a dosage of 375 mg/m^2^ weekly for 4 weeks, in line with common practice [6].

Daratumumab was administered as a compassionate-use therapy in patients with either with persistent ADAMTS-13 deficiency and detectable inhibitor despite rituximab or with a frequently relapsing disease course. The decision to begin daratumumab was not guided by predefined criteria but, rather, was according to physician’s choice. The standard daratumumab regimen consisted of intravenous infusions at a dose of 16 mg/kg body weight administered weekly over 6 weeks (1 patient received only 4 weekly doses during his first treatment course). One patient was treated with subcutaneous daratumumab at a fixed dose of 1800 mg weekly during induction, followed by monthly maintenance injections (Supplementary Table 1). Premedication included glucocorticoids, antihistamines, and acetaminophen. Adverse drug reactions were assessed according to Common Terminology Criteria for Adverse Events (version 5.0).

### 2.2 ADAMTS-13 activity and response

ADAMTS-13 activity and functional inhibitor titers were measured locally and centrally at the Swiss ADAMTS-13 reference laboratory at Bern University Hospital. Local testing by the participating centers was performed using different assay methods. As part of routine diagnostic quality control, plasma samples were stored frozen. For this retrospective study, available aliquots were reanalyzed centrally in Bern to ensure methodological consistency across the centers. Inhibitor titers were considered positive at ≥0.5 Bethesda Units (BU)/mL and capped at >2 BU/mL for reporting purposes.

Response criteria followed current and updated consensus guidelines [1,22]. Treatment response was categorized as partial and complete ADAMTS-13 recovery (≥20% to less than the lower limit of normal and equal to or more than the lower limit of normal corresponding to ≥50%, respectively). Persistent severe ADAMTS-13 deficiency was defined as an activity <10%. ADAMTS-13 activity measurements used to define treatment response were obtained after cessation of therapeutic plasma exchange and sufficiently distant from the last exchange to exclude interference from exogenous ADAMTS-13.

### 2.3 Ethical approval

The study was conducted in accordance with the Declaration of Helsinki. All patients provided written informed consent for off-label treatment with daratumumab and the publication of anonymized clinical data. The study was approved by the Ethics Committee of the Canton of Zürich, Switzerland (BASEC Nr. 2024-00276).

### 2.4 Statistical analysis

Data were entered into a digital case report form and analyzed using GraphPad Prism 10 (v10.2.3, GraphPad Software). Descriptive statistics are reported as median, mean, and range (minimum to maximum). ADAMTS-13 relapse-free survival was estimated using Kaplan-Meier analysis.

## 3 Results and Discussion

Using our outlined search strategy, we identified 5 patients with r/r iTTP who collectively received 8 treatment courses of daratumumab. Between January 2014 and May 2025, demographic and clinical data were collected, including comorbidities and prior therapies (summarized in Supplementary Table 1). Median age at initial iTTP diagnosis was 28 years (range, 19-32 years) and 32 years at initiation of daratumumab (range, 28-40 years). Patients received a median of 2 prior treatment regimens (range, 2-5), targeting both acute disease and ADAMTS-13 relapses. The median time from iTTP diagnosis to daratumumab initiation was 79 months (range, 4-139 months).

All patients had received rituximab prior to daratumumab. Three patients were treated with rituximab pre-emptively when ADAMTS-13 activity fell below 20%, and 2 in the context of a clinical relapse (Table). Daratumumab was initiated after a median of 21.2 weeks (range, 10.1-284.5 weeks) following rituximab initiation, therefore allowing for a potential delayed rituximab response (Supplementary Table 1). Two patients had a frequently relapsing course, while the remaining 3 experienced a treatment-refractory first episode. While no predefined criteria guided choice, daratumumab was administered in all cases to escalate immunosuppression when prior therapies had failed to eliminate pathogenic anti–ADAMTS-13 autoantibodies and restore ADAMTS-13 activity.

**Table:** Summary of daratumumab treatment parameters and associated clinical outcomes.

Parameter	Results
No. of daratumumab doses	6 (4-22)
Follow-up (mo)	44 (34-65)
Response rates	
Partial response (ADAMTS-13 activity 20%-50%)	0
Complete response (ADAMTS-13 activity >50%)	7
No response	1
ADAMTS-13 activity at response (%)	
Maximum	78 (53-102.2)
At 6 wk	62.2 (43-100)
Time to ADAMTS-13 response (d)	
Partial	13 (7-28)
Complete	26 (7-31)
ADAMTS-13 relapse following response to daratumumab	5
ADAMTS-13 relapse	3
Clinical relapse	2
Median time to subsequent treatment (mo)	19 (9-40)

Data are presented as median (range) or n.

ADAMTS-13, a disintegrin and metalloproteinase with thrombospondin type 1 motif 13.

Details on prior treatment regimens and outcomes are presented in the Table and Supplementary Table 2. A median of 6 daratumumab doses (range, 4-8) were administered per patient. One patient received ongoing monthly subcutaneous injections (1800 mg) following a 6-week induction phase.

Seven of 8 daratumumab treatment episodes (87.5%) resulted in ADAMTS-13 normalization, with a mean peak ADAMTS-13 activity of 80% (SD, ±20.3%). After previous complete responses to daratumumab, 1 episode (12.5%) failed to induce ADAMTS-13 recovery. This patient had been retreated with daratumumab twice due to frequent relapses, and the final course did not yield a therapeutic response.

**Figure 1:**
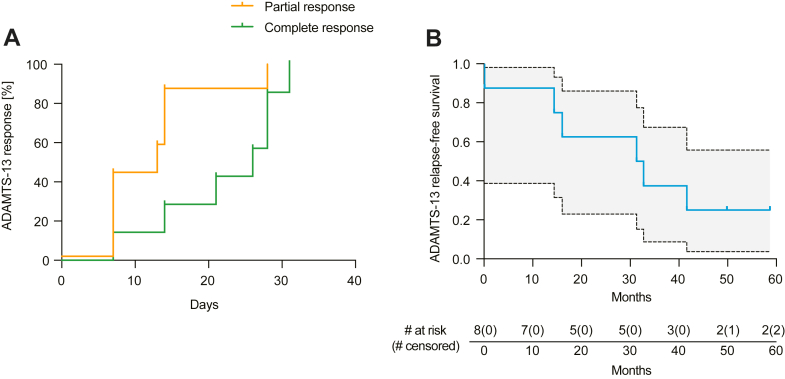
(A) Time from initiation of daratumumab to partial or complete a disintegrin and metalloproteinase with thrombospondin type 1 motif 13 (ADAMTS-13) response. (B) ADAMTS-13 relapse-free survival following response, defined as ADAMTS-13 activity ≥20%. Tick marks indicate censored observations. Numbers at risk are shown below the *x*-axis.

**Figure 2:**
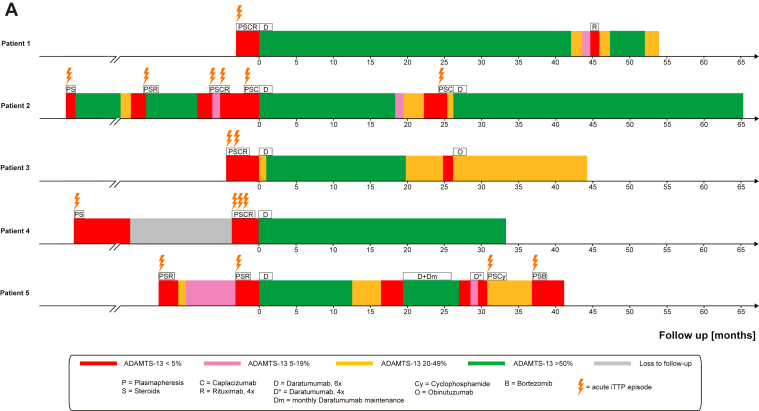
Timeline of treatment regimens, daratumumab administration, a disintegrin and metalloproteinase with thrombospondin type 1 motif 13 (ADAMTS-13) responses, and subsequent therapies. ADAMTS-13 response is graphed using a color-coded scale. Specific treatments are denoted using single letter abbreviations where appropriate. Clinical thrombotic thrombocytopenic purpura episodes are represented graphically by lightning bolts. The predaratumumab time period is not to scale. Bor, bortezomib; C, caplacizumab; Cy, cyclophosphamide; D, daratumumab, 6 doses; Dm, monthly daratumumab maintenance; D∗, daratumumab, 4 doses; O, obinutuzumab; P, therapeutic plasma exchange; R, rituximab; S, steroid.

In all responders, daratumumab rapidly eradicated the ADAMTS-13 inhibitor and restored ADAMTS-13 activity. Median times to partial and complete responses were 13 and 26 days, respectively (Figure 1A). After a median follow-up of 44 months (range, 34-65 months), the median ADAMTS-13 response duration (relapse-free survival) was 32 months (Figure 1B). At 12 months, 87.5% of patients remained in ADAMTS-13 remission, and median time to next treatment was 19 months (range, 9-40 months; Table 1). Four patients ultimately experienced ADAMTS-13 relapse following an initial response; re-exposure to daratumumab was successful in 2 of 3 retreatment episodes. At last follow-up, all patients were alive. Figure 2 illustrates the ADAMTS-13 response timelines. Notably, daratumumab was not only able to achieve durable remission in patients with frequently relapsing disease (Figure 2) but also remarkably effective in even those patients with severely refractory disease. Exposure to daratumumab in these cases lead to rapid eradication of ADAMTS-13 inhibitors (Supplementary Figure).

Our findings represent the longest follow-up reported to date in this setting. They are in line with previous reports [20,21,23], while observing a high complete response rate. In the largest published cohort, Weisinger et al. [21] reported 9 daratumumab treatment episodes in 7 patients pretreated with a median of 3 (range, 1-9) prior lines of immunosuppression. Partial and complete ADAMTS-13 remission occurred in 55% and 33% of episodes, respectively, with 1 refractory case, and were thus lower than in our cohort.

We observed no severe adverse reactions to daratumumab, particularly no infectious complications. We documented 2 mild to moderate infusion-related reactions (Common Terminology Criteria for Adverse Events grade 1-2), which resolved without sequelae.

The precise mechanism by which daratumumab exerts its effect in r/r iTTP remains incompletely understood. Plasmablasts and plasma cells likely drive persistent anti–ADAMTS-13 autoantibody production in some patients. An increased proportion of circulating plasmablasts was observed during early iTTP, with higher levels correlating with stronger inhibitory immunoglobulin G responses [24]. Conventional immunosuppressants such as glucocorticoids and B cell–depleting agents such as rituximab do not target long-lived plasma cells, which may persist for months or years. A similar persistence occurs in immune thrombocytopenia and other refractory autoantibody-mediated disorders [25].

ADAMTS-13 activity was monitored closely following daratumumab initiation, typically at least weekly during the early treatment phase (first 2 months). However, given the retrospective design, testing intervals were not fully standardized across patients. This may have introduced minor imprecision in the estimates of time to ADAMTS-13 response, which should be considered when interpreting early response kinetics.

In conclusion, daratumumab achieved rapid eradication of ADAMTS-13 inhibitors and restoration of ADAMTS-13 activity in 5 patients with r/r iTTP. Responses were durable, with ADAMTS-13 remission >12 months in most cases. We hypothesize that daratumumab, through selective targeting of pathogenic plasma cells, facilitates the clearance of inhibitory autoantibodies and recovery of ADAMTS-13 activity. These findings support daratumumab as a safe and effective treatment option in patients with r/r iTTP who fail to respond to or relapse after rituximab.

## References

[1] Joly B.S., Coppo P., Veyradier A. (2017). Thrombotic thrombocytopenic purpura. Blood.

[2] Coppo P., Bubenheim M., Benhamou Y., Völker L., Brinkkötter P., Kühne L. (2025). Caplacizumab use in immune-mediated thrombotic thrombocytopenic purpura: an international multicentre retrospective Cohort study (The Capla 1000+ project). EClinicalMedicine.

[3] Dutt T., Shaw R.J., Stubbs M., Yong J., Bailiff B., Cranfield T. (2021). Real-world experience with caplacizumab in the management of acute TTP. Blood.

[4] Zheng X.L., Vesely S.K., Cataland S.R., Coppo P., Geldziler B., Iorio A. (2020). ISTH guidelines for treatment of thrombotic thrombocytopenic purpura. J Thromb Haemost.

[5] Scully M., Cataland S.R., Peyvandi F., Coppo P., Knöbl P., Kremer Hovinga J.A. (2019). Caplacizumab treatment for acquired thrombotic thrombocytopenic purpura. N Engl J Med.

[6] Owattanapanich W., Wongprasert C., Rotchanapanya W., Owattanapanich N., Ruchutrakool T. (2019). Comparison of the long-term remission of rituximab and conventional treatment for acquired thrombotic thrombocytopenic purpura: a systematic review and meta-analysis. Clin Appl Thromb Hemost.

[7] Jestin M., Benhamou Y., Schelpe A.S., Roose E., Provôt F., Galicier L. (2018). Preemptive rituximab prevents long-term relapses in immune-mediated thrombotic thrombocytopenic purpura. Blood.

[8] Kremer Hovinga J.A., Vesely S.K., Terrell D.R., Lämmle B., George J.N. (2010). Survival and relapse in patients with thrombotic thrombocytopenic purpura. Blood.

[9] Ahmad H.N., Thomas-Dewing R.R., Hunt B.J. (2007). Mycophenolate mofetil in a case of relapsed, refractory thrombotic thrombocytopenic purpura. Eur J Haematol.

[10] Bichard C., Mancini I., Agosti P., Capecchi M., De Leo P., Arcudi S. (2022). Efficacy and safety of azathioprine during remission of immune-mediated thrombotic thrombocytopenic purpura. Blood Adv.

[11] Beloncle F., Buffet M., Coindre J.P., Munoz-Bongrand N., Malot S., Pène F. (2012). Splenectomy and/or cyclophosphamide as salvage therapies in thrombotic thrombocytopenic purpura: the French TMA Reference Center experience. Transfusion.

[12] Kierdorf H., Maurin N., Heintz B. (1993). Cyclosporine for thrombotic thrombocytopenic purpura. Ann Intern Med.

[13] Patriquin C.J., Thomas M.R., Dutt T., McGuckin S., Blombery P.A., Cranfield T. (2016). Bortezomib in the treatment of refractory thrombotic thrombocytopenic purpura. Br J Haematol.

[14] Doyle A.J., Stubbs M.J., Lester W., Thomas W., Westwood J.P., Thomas M. (2022). The use of obinutuzumab and ofatumumab in the treatment of immune thrombotic thrombocytopenic purpura. Br J Haematol.

[15] Kremer Hovinga J.A., Studt J.D., Demarmels Biasiutti F., Solenthaler M., Alberio L., Zwicky C. (2004). Splenectomy in relapsing and plasma-refractory acquired thrombotic thrombocytopenic purpura. Haematologica.

[16] Schaller M., Vogel M., Kentouche K., Lämmle B., Kremer Hovinga J.A. (2014). The splenic autoimmune response to ADAMTS13 in thrombotic thrombocytopenic purpura contains recurrent antigen-binding CDR3 motifs. Blood.

[17] Mahévas M., Patin P., Huetz F., Descatoire M., Cagnard N., Bole-Feysot C. (2013). B cell depletion in immune thrombocytopenia reveals splenic long-lived plasma cells. J Clin Invest.

[18] Ejaz K., Roback J.D., Stowell S.R., Sullivan H.C. (2021). Daratumumab: beyond multiple myeloma. Transfus Med Rev.

[19] Van den Berg J., Kremer Hovinga J.A., Pfleger C., Hegemann I., Stehle G., Holbro A. (2022). Daratumumab for immune thrombotic thrombocytopenic purpura. Blood Adv.

[20] Aggarwal A., White D., Pavord S., Thomas W., Desborough M.J.R. (2023). Daratumumab for refractory immune-mediated thrombotic thrombocytopenic purpura. Br J Haematol.

[21] Weisinger J., Bouzid R., Ranta D., Woaye-Hune P., Cohen-Aubart F., Gaible C. (2024). Efficacy and safety of daratumumab in multiresistant immune-mediated thrombotic thrombocytopenic purpura. Br J Haematol.

[22] Cuker A., Cataland S.R., Coppo P., de la Rubia J., Friedman K.D., George J.N. (2021). Redefining outcomes in immune TTP: an international working group consensus report. Blood.

[23] Xie X.T., Xiao Y.Y., Zhang Y., Luo Z.M., Luo Y. (2023). Combination regimens containing daratumumab for initial diagnosed acquired thrombotic thrombocytopenic purpura. J Thromb Thrombolysis.

[24] Shin J.S., Subhan M.O., Cambridge G., Guo Y., de Groot R., Scully M. (2022). Alterations in B- and circulating T-follicular helper cell subsets in immune thrombotic thrombocytopenic purpura. Blood Adv.

[25] Chen M., Shortt J. (2022). Plasma cell directed therapy for immune thrombotic thrombocytopenic purpura (iTTP). Transfus Med Rev.

