# Supplementary material for: Daratumumab for relapsed refractory immune thrombotic thrombocytopenic purpura: initial response and long-term follow-up
Source: Res Pract Thromb Haemost. 2026 Mar 4;10(2):103405. doi: 10.1016/j.rpth.2026.103405 (PMC13131128; doi:10.1016/j.rpth.2026.103405)
Supplement: Supplementary Material [file mmc1.docx]

**Supplementary Table 1.** Baseline demographic characteristics, comorbidities, and prior treatment history of included patients.

|  | **Patient 1**^19^ | **Patient 2**^19^ | **Patient 3** | **Patient 4** | **Patient 5** |
| --- | --- | --- | --- | --- | --- |
| **Age at iTTP diagnosis (years)** | 31 | 26 | 32 | 19 | 28 |
| **Age at initial daratumumab dose** | 31 | 32 | 32 | 28 | 40 |
| **Sex** | Female | Male | Female | Female | Male |
| **Ethnicity** | Caucasian | Caucasian | Caucasian | Caucasian | Caucasian |
| **Relevant comorbidities or pregnancy** | Hypothyroidism, Pregnancy | none | none | Pregnancy | Metabolic syndrome  HOCM |
| **Daratumumab treatment episodes (doses per episode)** | 1 (6) | 2 (6, 6) | 1 (6) | 1 (6) | 3 (6, 6, 4) |
| **Daratumumab maintenance therapy** | No | No | No | No | Yes |
| **Time from last rituximab to daratumumab (weeks)** | 10.1 | 23.5 | 13.0 | 21.2 | 28.4 |
| **Prior iTTP episodes** | 0 | 2 | 1 | 3 | 2 |
| **Median no. of prior treatment lines per patient** | 2 | 5 | 2 | 4 | 2 |
| **Clinical course of iTTP** | Primary refractory | Frequently relapsing | Primary refractory | Primary refractory | Frequently relapsing |
| **Treatment duration (doses)**  **TPE**  **Caplacizumab**  **Rituximab**  **Obinutuzumab**  **Daratumumab** | 15  121  4  0  6 | 22  30  8  0  12 | 11  37  8  1  6 | 24  30  4  0  6 | 23  0  6  1  22 |
| **Adverse events attributed to daratumumab** | Yes (grade 1) | Yes (grade 2) | No | No | No |

**Supplemental Table 2.** Longitudinal ADAMTS13 activity levels and corresponding clinical responses to daratumumab therapy.

|  | **Patient 1** | **Patient 2** | **Patient 3** | **Patient 4** | **Patient 5** |
| --- | --- | --- | --- | --- | --- |
| **ADAMTS13 at daratumumab initiation**  Activity  Inhibitor | < 5%  > 2 BU/mL | < 5%  > 2 BU/mL | < 5%  > 2 BU/mL | < 5%  > 2 BU/mL | < 5%  > 2 BU/mL |
| **Maximal ADAMTS13 activity**  **following daratumumab**  Activity  Inhibitor | 99%  0 BU/mL | 102.2%  0 BU/mL | 53%  0 BU/mL | 77.8%  N/A | 58% and 62%  0 BU/mL |
| **Relapse**  Months after daratumumab | ADAMTS13 relapse  41 months | (after 1^st^ Dara exposure), clinical relapse  32 months | ADAMTS13 relapse  29 months | None | ADAMTS13 relapse and clinical relapse, resp.  12 and 8 months, resp. |

**Supplementary Figure and Table Legends**

**Supplementary Table 1.**

Summary of demographic characteristics, relevant comorbidities, and prior therapies.

*Abbreviations*: ADAMTS13, A Disintegrin And Metalloproteinase with ThromboSpondin-1 motifs, 13; TPE, therapeutic plasma exchange; GC, glucocorticoids; HOCM, hypertrophic obstructive cardiomyopathy.

**Supplementary Table 2.**

ADAMTS13 activity and treatment response following daratumumab.

*Abbreviation*: ADAMTS13, A Disintegrin And Metalloproteinase with ThromboSpondin-1 motifs, 13.

**Supplementary Figure 1.**

Clinical course and selected laboratory parameters in patient 1 with refractory iTTP, from initial hospital admission to latest follow-up. Red line: ADAMTS13 inhibitor titer (Bethesda units [BU] per mL; truncated at 2 BU/mL). Blue line: ADAMTS13 activity (%). Grey line: platelet count (x10^9^ /L).
