# Supplementary figures and images for: Daratumumab for relapsed refractory immune thrombotic thrombocytopenic purpura: initial response and long-term follow-up
Source: Res Pract Thromb Haemost. 2026 Mar 4;10(2):103405. doi: 10.1016/j.rpth.2026.103405 (PMC13131128; doi:10.1016/j.rpth.2026.103405)

Supplemental Figure 1

A

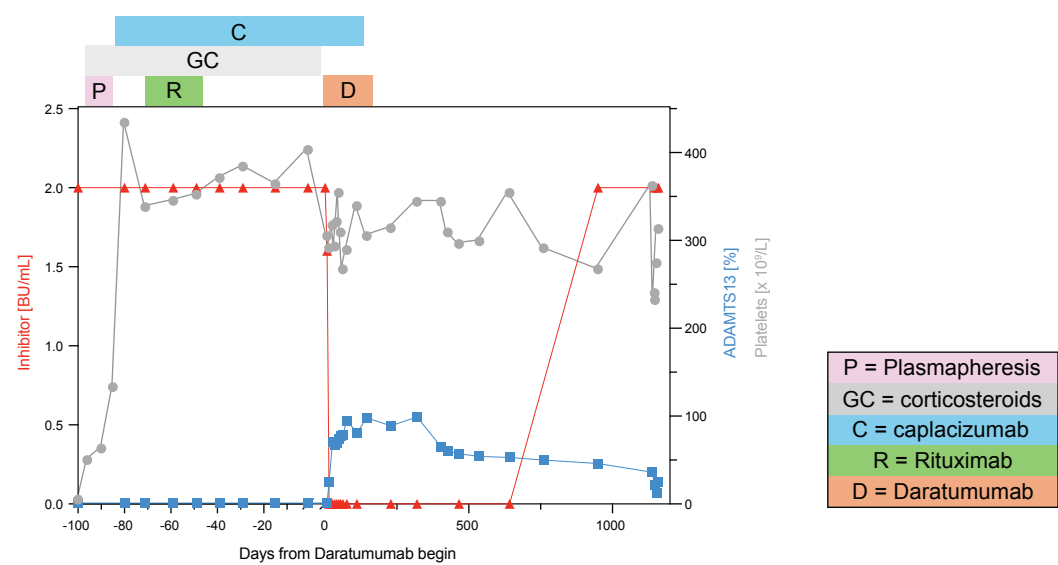

Supplement: Supplementary Figure 1 [file mmc2.pdf]
